# INFLATE: a protocol for a randomised controlled trial comparing nasal balloon autoinflation to no nasal balloon autoinflation for otitis media with effusion in Aboriginal and Torres Strait Islander children

**DOI:** 10.1186/s13063-022-06145-8

**Published:** 2022-04-14

**Authors:** Robyn Walsh, Jennifer Reath, Hasantha Gunasekera, Amanda Leach, Kelvin Kong, Deborah Askew, Federico Girosi, Wendy Hu, Timothy Usherwood, Sanja Lujic, Geoffrey Spurling, Peter Morris, Chelsea Watego, Samantha Harkus, Cheryl Woodall, Claudette Tyson, Letitia Campbell, Sylvia Hussey, Penelope Abbott

**Affiliations:** 1 School of Medicine, Western Sydney University, Sydney, Australia; 2 Faculty of Medicine and Health, The University of Sydney, Sydney, Australia; 3 Menzies School of Health Research, Darwin, Australia; 4 School of Medicine and Public Health, The University of Newcastle, Newcastle, Australia; 5 Faculty of Medicine, The University of Queensland, Brisbane, Australia; 6 Centre for Big Data Research in Health, The University of New South Wales, Sydney, Australia; 7 Southern Queensland Centre of Excellence in Aboriginal and Torres Strait Islander Primary Health Care (Inala Indigenous Health Service) Queensland Health, Brisbane, Australia; 8 Faculty of Health, Queensland University of Technology, Brisbane, Australia; 9 Hearing Australia, Sydney, Australia; 10 Tharawal Aboriginal Corporation, Sydney, Australia; 11 Kalwun Development Corporation, Gold Coast, Australia; 12 Townsville Aboriginal and Islander Health Service, Townsville, Australia

**Keywords:** Otitis media, Otitis media with effusion, Aboriginal and Torres Strait Islander, Indigenous, Children, Nasal autoinflation, Randomised controlled trial, Ear disease, Otolaryngology

## Abstract

**Background:**

Otitis media with effusion (OME) is common and occurs at disproportionately higher rates among Indigenous children. Left untreated, OME can negatively affect language, development, learning, and health and wellbeing throughout the life-course. Currently, OME care includes observation for 3 months followed by consideration of surgical ventilation tube insertion. The use of a non-invasive, low-cost nasal balloon autoinflation device has been found beneficial in other populations but has not been investigated among Aboriginal and Torres Strait Islander children.

**Methods/design:**

This multi-centre, open-label, randomised controlled trial will determine the effectiveness of nasal balloon autoinflation compared to no nasal balloon autoinflation, for the treatment of OME among Aboriginal and Torres Strait Islander children in Australia. Children aged 3–16 years with unilateral or bilateral OME are being recruited from Aboriginal Health Services and the community. The primary outcome is the proportion of children showing tympanometric improvement of OME at 1 month. Improvement is defined as a change from bilateral type B tympanograms to at least one type A or C1 tympanogram, or from unilateral type B tympanogram to type A or C1 tympanogram in the index ear, without deterioration (type A or C1 to type C2, C3, or B tympanogram) in the contralateral ear. A sample size of 340 children (170 in each group) at 1 month will detect an absolute difference of 15% between groups with 80% power at 5% significance. Anticipating a 15% loss to follow-up, 400 children will be randomised. The primary analysis will be by intention to treat. Secondary outcomes include tympanometric changes at 3 and 6 months, hearing at 3 months, ear health-related quality of life (OMQ-14), and cost-effectiveness. A process evaluation including perspectives of parents or carers, health care providers, and researchers on trial implementation will also be undertaken.

**Discussion:**

INFLATE will answer the important clinical question of whether nasal balloon autoinflation is an effective and acceptable treatment for Aboriginal and Torres Strait Islander children with OME. INFLATE will help fill the evidence gap for safe, low-cost, accessible OME therapies.

**Trial registration:**

Australia New Zealand Clinical Trials Registry ACTRN12617001652369. Registered on 22 December 2017. The Australia New Zealand Clinical Trials Registry is a primary registry of the WHO ICTRP network and includes all items from the WHO Trial Registration data set. Retrospective registration.

**Supplementary Information:**

The online version contains supplementary material available at 10.1186/s13063-022-06145-8.

## Introduction

### Background

Otitis media with effusion (OME) is a common health condition among Aboriginal and Torres Strait Islander children in Australia with limited treatment options. This clinical trial investigates the effectiveness of nasal balloon autoinflation, a relatively inexpensive and readily available treatment for OME.

#### Diagnosis and incidence of otitis media with effusion

Otitis media with effusion, one of several inflammatory middle ear diseases collectively known as otitis media (OM), is characterised by accumulated fluid (effusion) and reduced air pressure in the middle ear space [1]. It is diagnosed by otoscopy and tympanometry [2].

**Table:** 

**Tympanometry:** Best practice guidelines [2, 3] recommend pneumatic otoscopy and tympanometry for the diagnosis of OME. Tympanometry is a non-invasive test measuring tympanic membrane movement and middle ear air pressure — a proxy for determining the presence of middle ear effusion — and is depicted in graphical form (tympanogram). Tympanograms are classified as type A, B, C1, C2, or C3. Peaked type A and C1 tympanograms indicate the absence of effusion consistent with normal middle ears. A flat type B trace is diagnostic of middle ear effusion in the absence of pain and/or a bulging or perforated tympanic membrane. Type C2 and C3 peaked traces are characterised by significant negative middle ear pressure and are considered equivocal and cannot be used to either diagnose or exclude OME [4, 5].	

Acute otitis media (AOM) is a frequent reason for children to attend general practice and often results in the prescription of antibiotics [6, 7]. Resolution of AOM is often followed by a period of residual middle ear fluid, or OME. The prevalence of OME in Aboriginal and Torres Strait Islander children is disproportionately high, ranging from 10 to 30% [8, 9]. The risk factors are multifactorial including household crowding and poor functionality [10], exposure to tobacco smoke, food insecurity, child care attendance, older siblings, upper respiratory tract infection, family history, lack of breastfeeding [8], and socioeconomic disadvantage and other adverse social determinants of health arising as a consequence of ongoing colonisation [11, 12]. The duration and severity of disease, and the incidence of complications, are also higher among Aboriginal and Torres Strait Islander people, particularly in remote and rural regions of Australia [13, 14].

#### Prognosis and treatment of otitis media with effusion

In the absence of medical intervention, 30% of OME cases in children resolve within the first month of diagnosis, and 90% within 3 months of diagnosis, as determined by international longitudinal research [15]. Although most cases resolve spontaneously by 3 months, recurrent episodes and persistent OME can result in impaired hearing and delay in language development with a negative impact on education and wellbeing [16–18]. All forms of OM, including OME, are associated with hearing loss, increasing in severity with more severe forms of OM [19].

Treatment options for episodic, unilateral, or persistent OME are limited. Treatment with antibiotics is of minimal benefit, and not without risk, for improving resolution of OME, hearing, or reducing tympanostomy tube insertion [20], while nasal decongestants and steroids are of no benefit [21, 22]. Guidelines for OM in Aboriginal and Torres Strait Islander children recommend that children with persistent bilateral OME for 3 months are referred to an audiologist for a diagnostic hearing assessment and to an Ear Nose and Throat surgeon (ENT) to consider insertion of tympanostomy tube(s) to re-establish normal middle ear pressure. This enables clearing of the middle ear effusion and aeration of the middle ear space [21], thereby reducing the risk of longer term hearing loss and other complications. However, there are temporal and financial costs and health risks associated with this approach. The deferral of surgical tympanostomy tube insertion to at least 3 months after initial diagnosis means no active treatment and a potential decline in hearing in children with persistent OME, during this period. Additionally, access to surgery is extremely limited for many Aboriginal and Torres Strait Islander children with OM living in rural and remote areas where surgical wait lists can be years [23]. Furthermore, although unilateral OME is increasingly being recognised as contributing to adverse outcomes including spacial processing disorder [24], guideline-recommended treatment approaches for unilateral disease based on high-quality intervention studies are lacking.

Surgical tympanostomy tube insertion (with or without adenoidectomy) is costly [7, 25]. Surgical intervention is also limited in its long-term effectiveness [26] and has been shown to result in a 26–37% incidence of post-tympanostomy infection [27, 28]. There is a need for an immediate, low-risk, cost-effective, and easy-to-use alternative treatment for both bilateral and unilateral OM. Nasal balloon autoinflation may be useful immediately upon diagnosis of bilateral or unilateral OME.

#### Nasal balloon autoinflation

Nasal balloon autoinflation is designed to introduce air into the middle ear and facilitate fluid drainage. It can be performed by a person with OME, in any location, potentially offering a possible low-cost, low-risk, readily accessible, and easy-to-use alternative to ventilation tube surgery [29].

The effects of nasal balloon autoinflation on OME, measured using tympanometry and audiometry, were examined in a 2013 Cochrane review consisting of a pooled analysis of 8 small trials (7/8 studies with ≤100 participants) [30]. No statistically significant difference was shown for tympanometry changes; however, a composite measure of tympanometric or audiometric improvement showed nasal balloon autoinflation for OME was effective at more than 1 month, compared to no nasal balloon autoinflation (relative risk increase = 1.74; 95% confidence interval 1.22 to 2.50). Treatment adherence varied from 43 to 98%. There was no significant difference in adverse events between nasal balloon autoinflation and no nasal balloon autoinflation, with one study documenting ear pain with balloon autoinflation. The authors interpreted the findings as ‘favourable’ but noted that the included studies were small, and recommended further research in primary care. A subsequent study published in 2015 of 320 children (4–11 years) with unilateral and bilateral OME showed tympanometric resolution at 3 months (adjusted relative risk = 1.37, 95% confidence interval 1.03 to 1.83) in a nasal balloon autoinflation group versus no nasal balloon autoinflation group [31]. The study also reported improved ear health-related quality of life and good adherence to treatment (80% at 3 months). Another study of 45 children demonstrated improvement in hearing thresholds after 1 month (difference = dB. 95% CI) and concluded the device could be useful while children were on waiting lists for surgical treatment [32]. Although Aboriginal and Torres Strait Islander children are at increased risk of OM complications, no studies of nasal balloon autoinflation have been undertaken in this population.

## Methods/design

INFLATE is a pragmatic, multi-centre, two-arm, open-label, parallel design, superiority, randomised controlled trial comparing nasal balloon autoinflation to no nasal balloon autoinflation for the treatment of OME among Aboriginal and Torres Strait Islander children.

### Aim

The INFLATE trial aims to investigate whether, among Aboriginal and Torres Strait Islander children aged 3 to 16 years with unilateral or bilateral OME, nasal balloon autoinflation increases the resolution of middle ear effusion compared with no nasal balloon autoinflation based on tympanometry.

*Null hypothesis (H0)*: The proportion of children with tympanometric improvement at 1 month from baseline will be equal in the nasal autoinflation and no nasal autoinflation (usual care) groups.

*Alternative hypothesis (H1)*: The proportion of children with tympanometric improvement at 1 month from baseline will be less in the nasal autoinflation treatment group compared to the usual care treatment group.

### Primary objective

To determine whether, among Aboriginal and Torres Strait Islander children aged 3 to 16 years with OME, nasal balloon autoinflation compared to no nasal balloon autoinflation improves unilateral and bilateral OME determined by tympanometric findings at 1 month from baseline.

### Secondary objectives

1. Determine whether nasal balloon autoinflation compared to no nasal balloon autoinflation
  - Improves unilateral and bilateral OME determined by tympanometric findings at 3 and 6 months from baseline
  - Reduces hearing loss at 3 months from baseline
  - Improves ear-related health and quality of life at 3 and 6 months from baseline
2. Determine
  - Adverse events up to 1, 3, and 6 months from baseline
  - Parent/carer/child reported adherence to nasal balloon autoinflation
  - Cost-effectiveness of nasal balloon autoinflation for the management of OME
  - Parent/carer, health care provider, Aboriginal Health Service Research Officer, and community reference group views regarding nasal balloon autoinflation acceptability and use, OME and its management, research in Aboriginal Health Services, and the implementation and receipt of the trial

### Setting

The trial is being conducted in urban and regional Aboriginal and Torres Strait Islander primary health care services across Australia (New South Wales, Queensland, the Australian Capital Territory, and Victoria). The services include Aboriginal community-controlled health services governed by local Aboriginal communities and one state government-run Aboriginal and Torres Strait Islander primary health care facility. The Aboriginal Health Service staff are multidisciplinary, including dedicated Aboriginal Health Workers and often contracted services providing ear and hearing health care. At the time of initiation of INFLATE, some services were already participating in the WATCH trial, a clinical trial of the management of acute otitis media [33]. The two trials have shared governance, implementation of recruitment, and staff.

### Governance, mentorship, and training

A Steering Committee, Operational Management Group, a Data Safety Monitoring Board (DSMB), and other working groups consisting of chief investigators (CIs), associate investigators (AIs), and Aboriginal Health Service research staff meet regularly to ensure the protocol is implemented correctly, recruitment is monitored, issues are addressed as they arise, and to develop, refine, implement, and document trial materials and processes. All Aboriginal Health Services are encouraged to establish Aboriginal and Torres Strait Islander community reference groups or equivalent structures. Consultation and communication with and between each service and their community are conducted regularly to review local implementation of the trial, maintain community engagement, and regularly disseminate trial findings to the community. Management of the trial between investigators and Aboriginal Health Services is a non-hierarchical partnership, recognising the different expertise, experiences, and knowledge necessary to successfully conduct research in Aboriginal and Torres Strait Islander primary health care and facilitate knowledge transfer.

Aboriginal and/or Torres Strait Islander researchers and local community reference groups provide input into and feedback from trial implementation in the Aboriginal and Torres Strait Islander primary health care setting and assist other team members to ensure all stakeholders remain respectful towards and learn from participating Aboriginal and Torres Strait Islander communities. Community reference group members are remunerated for their time during 6–12 monthly community reference group meetings while health services are remunerated for patient recruitment and follow-up in recognition of General Practitioner (GP) time.

Service staff and local communities are building research and ear health-related skills, knowledge, and experience. Service staff are regularly trained in research practices and the use of pneumatic otoscopy and tympanometry to detect middle ear effusion, during service initiation and monitoring visits, at biannual face-to-face investigator meetings, and via regular trial emails and newsletters. Concurrently, Western Sydney University (WSU) staff are building their understanding of best practice in the implementation of clinical trials in the Aboriginal Health Service setting.

### Study population/eligibility criteria

Aboriginal or Torres Strait Islander children, attending urban or regional Aboriginal Health Services, aged 3–16 years with unilateral or bilateral type B tympanograms are eligible for inclusion. Tympanometry is conducted by a trained research officer (RO), and eligibility, including trace type, is confirmed by the treating General Practitioner (GP). Exclusion criteria include clinical confirmation of a current acute upper respiratory infection, acute otitis media, perforated tympanic membrane, or ventilation tube in situ; ear surgery booked within 1 month of diagnosis; self-reported latex allergy, recent nosebleed (at least 1 nosebleed in last 3 weeks or > 1 nosebleeds in last 6 months); self-reported or documented history of an at-risk condition (e.g., immunosuppression); previous participation in the trial; or self-reported inability to use the autoinflation device (demonstrated by the inability to blow nose).

### Recruitment strategies

Recruitment strategies include a review of existing health service clinical databases for patients with a history of ear disease, opportunistic screening of children visiting health services, school ear screenings and community health promotion events, advertising via promotional posters and flyers in all health service waiting rooms and at health promotion events, and providing regular trial updates to health service staff and community reference groups via meetings, newspaper articles, and periodic emails and newsletters.

### Screening/enrolment

Aboriginal Health Services are funded to recruit research officers (ROs) who, with GPs and other health care providers, identify potential participants by reviewing existing health service clinical databases and medical records for children with past and/or current OM. Treating clinicians or their delegate then contact a child’s parent/carer via phone or mail to inform them about the trial and assess their interest in participating. Children of interested parent/carers are invited to attend the health service for formal screening and confirmation of eligibility. Research officers, GPs, and other health care providers also opportunistically screen all children visiting Aboriginal Health Services regardless of the reason for attendance, and at school ear screenings and community health promotion events and, if found eligible, formal screening is offered to confirm eligibility. Children are considered enrolled where eligibility is confirmed by a GP and written informed consent to participate is obtained. Randomisation and baseline assessments are conducted immediately following enrolment (day 0).

### Treatment groups

The intervention group uses a nasal balloon autoinflation device, Otovent® [34], in both nostrils three times daily from baseline (day 0) for up to 3 months. Participants are instructed to temporarily cease autoinflation treatment while symptomatic for no more than 5 consecutive days in the event of an upper respiratory tract infection to minimise the potential for ear pain during autoinflation. Participants cease autoinflation at 1 month if they have bilateral type A, C1, C2, or C3 tympanograms. Those with a type B tympanogram in at least one ear are asked to continue autoinflation for a further 2 months. At 3 months, participants are advised to cease autoinflation irrespective of tympanogram results.

The control group is managed according to Australian treatment guidelines (usual care) and regular follow-up with ROs, without autoinflation. General Practitioners decide usual care taking into consideration a participant’s risk of more severe forms of OM.

Antibiotics, steroids, antihistamines, decongestants, and alternative therapies are not recommended use during trial participation. All children in the intervention and control groups undergo a hearing assessment by an audiologist at either the Aboriginal Health Service or an audiology service provider. Any participants with bilateral OME or hearing impairment at 3 months are reviewed by a GP for appropriate management, including referral for ENT assessment.

### Randomisation

Participants are randomised to the intervention or control group by the RO using an automated Interactive Voice Response System (IVRS) located at the National Health and Medical Research Council Clinical Trials Centre (NHMRC CTC), Sydney, Australia. The IVRS, accessible by telephone, uses a computer-generated randomisation sequence, enabling immediate 1:1 allocation to unblinded treatment groups. Participants and health service and WSU staff are not blinded to treatment allocation due to the nature of the intervention. Randomisation is stratified using permuted block design (block size 4), based on participating site, child age (3 to 5 years versus 6 to 16 years), and whether unilateral or bilateral OME at presentation. These strata were determined a priori given their potential association with effectiveness.

### Follow-up (visits/assessments/measures/tools)

**Fig. 1:**
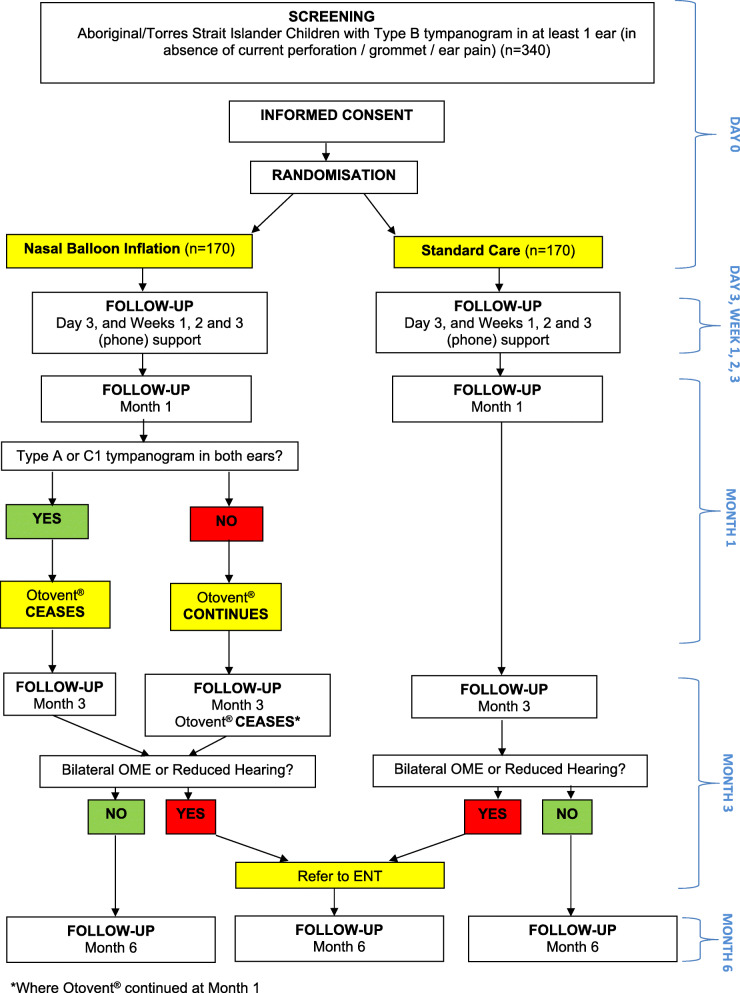
Recruitment flowchart

Follow-up (Fig. 1) by the RO occurs via phone at day 3 and weekly from weeks 1–3 and 5–11 and at Aboriginal Health Services at 1, 3, and 6 months post-enrolment and randomisation (day 0). Data sources, the findings of which are recorded on trial-designed paper source document worksheets, include tympanometry, video pneumatic otoscopy, audiometry, adherence and cost-effectiveness progress questions, an OMQ-14 quality of life questionnaire [35], nasal autoinflation adherence diary, and qualitative interviews. Medically trained CIs review participant medical records for additional information related to ear disease and treatment including adverse events, health care utilisation, and medication usage, for up to 6 months post-randomisation. This information informs determination of secondary outcomes including the cost-effectiveness analysis. Details of data collection at each of the scheduled assessment times are summarised in Table 1.

**Table 1:**
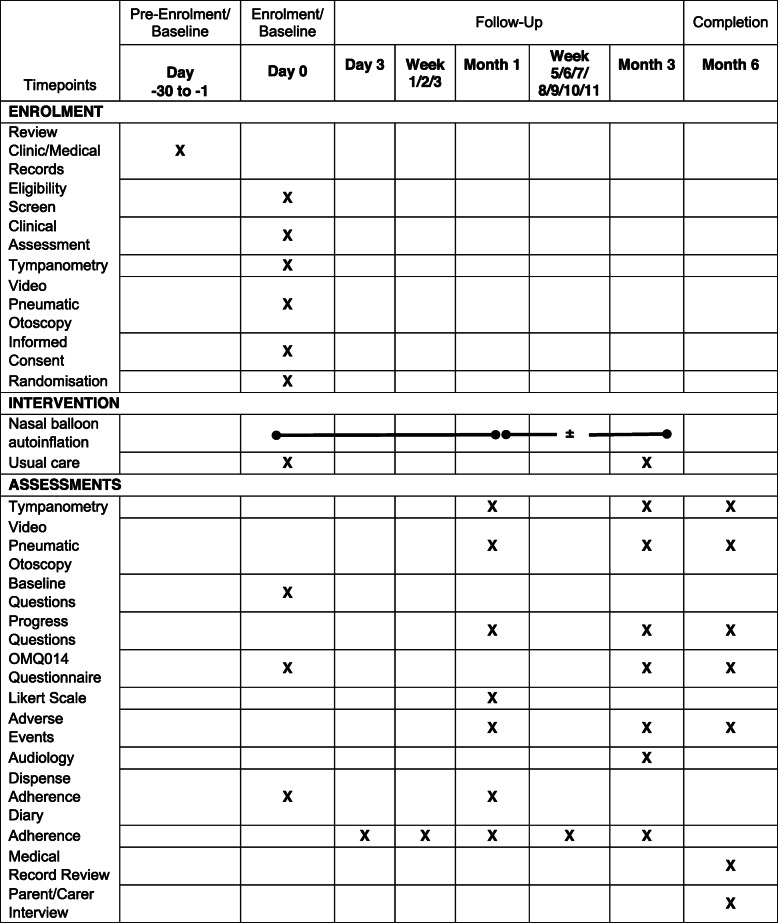
Enrolment/intervention/assessments

This trial does not include collection of biological specimens for current or future use.

### Study completion/withdrawal/lost to follow-up

Study completion is defined as a completion of 1- and 6-month assessments at minimum. Participants may withdraw or be withdrawn from the trial by a parent/carer, treating physician, or CIs at any stage of follow-up. The independent DSMB may also recommend participant withdrawal from the trial in the event of a significant safety issue. Reasons for and the extent of withdrawal (treatment only, treatment and trial assessments, or treatment, trial assessments, and data collection) are recorded. Participants who have not withdrawn and do not complete either their 1- or 6-month assessment are deemed lost to primary outcome or final status follow-up respectively.

### Data management

Source data is captured either in password-protected participant electronic medical record databases, on trial-designed paper source document worksheets, or diagnostic device paper or electronic software output. Paper source documents are stored in a lockable cabinet, and electronic source documents in password-protected health service databases, accessible to authorised health service staff only. Source data is de-identified and coded using randomisation number before it is single entered by the RO into an encrypted, password-protected validated electronic Case Report Form database (Medrio™) hosted in the USA. De-identified, coded data is accessible to authorised health service and WSU staff only. Qualitative interviews are taped and transcribed, then electronic copies de-identified and coded using randomisation number, for storing by WSU. Paper and electronic source data will be securely archived by health services, and de-identified, coded data, by WSU, for a minimum of 15 years after completion of the trial.

### Data monitoring

All data collected from the first patient recruited at each participating site are reviewed for accuracy and validity. Consent, eligibility, primary outcome data points, serious adverse events, and select adverse events of interest are monitored for all participants. All remaining data are randomly monitored (estimated 10% of participants) for quality assurance purposes.

INFLATE is a low-risk trial using the nasal autoinflation device within its marketed indication and population. Given the low-risk nature of the trial, the use of ongoing centralised statistical and on-site monitoring, and ongoing site and WSU staff training, an external audit will not be conducted.

### Sample size and power

It is estimated that a sample size of 340 children (170 in each treatment group) will detect an absolute difference of 15% between groups (relative difference relative risk = 1.43) in OME resolution rates at 1 month, with 80% power and a 5% significance level. The 15% difference represents a difference between an estimated 35% resolution in the control group and 50% resolution in the treatment group based on a similar trial [36]. This 15% absolute difference reduces the 65% non-resolution by approximately a quarter, which is clinically relevant. Assuming a 15% loss to follow-up, 400 children will need to be randomised. This sample size will also detect an effect size of 0.3 (assuming a standardised standard deviation = 1) for continuous secondary outcomes, such as OMQ-14 scores.

### Outcomes

#### Primary outcome

The proportion of children showing tympanometric improvement of OME at 1 month from baseline. Improvement is defined as a change from bilateral type B tympanograms to at least one type A or C1 tympanogram, or from unilateral type B tympanogram to type A or C1 tympanogram in the index ear, without deterioration (type A or C1 to type C2, C3, or B tympanogram) in the contralateral ear.

#### Secondary outcomes

The secondary outcomes are:

i) Proportion of children showing tympanometric improvement at 3 and 6 months from baseline (OME improved by child)
ii) Proportion of ears showing tympanometric resolution of OME at 1, 3, and 6 months from baseline
iii) Proportion of children showing tympanometric improvement at 1, 3, and 6 months from baseline (OME improved by child — unilateral and bilateral subgroups)
iv) Proportion of tympanometric deterioration from a type A or C1 tympanogram to C2, C3, or B tympanogram in non-index ears at 1, 3, and 6 months from baseline (new OME by ear — unilateral subgroup)
v) 3-frequency average hearing levels (pure tone threshold audiometry) at 3 months from baseline
vi) Ear-related health and quality of life score (using OMQ-14 quality of life questionnaire) at day 0 and months 3 and 6
vii) Adverse events up to 6 months from baseline
viii) Adherence to autoinflation from day 0 to month 1 (using paper adherence diary)
ix) Medical and non-medical costs of OME and its treatment
x) Qualitative exploration of parent/carer and health care provider, AMS Research Officer and AMS reference group perspectives of otitis media, research processes and the research experience including the experience of using nasal balloon autoinflation

##### Adverse events

Only adverse events meeting the standard international [37] definition of serious, or events of interest related to otitis media or nasal balloon autoinflation, solicited and via medical records, are recorded. Serious adverse events are defined as death, are immediately life-threatening, cause permanent incapacitation, result in hospitalisation or prolongation of hospitalisation, or are medically important events. Adverse events of interest related to otitis media or nasal balloon autoinflation include upper respiratory tract infection, ear discharge, tonsillitis, ear pain, hearing problems, dizziness, nose bleed, and latex allergy. Non-serious adverse events defined per protocol as unrelated to OM or nasal balloon autoinflation are not recorded. Serious adverse events and adverse events of interest are reported 6–12monthly at DSMB meetings. At final data analysis, adverse events will be coded using the International Statistical Classification of Diseases and Related Health Problems, Tenth Revision, Australian Modification 11^th^ Edition (ICD-10-AM, 11th edition) [38], and code totals tallied and tabulated, and percentages compared according to treatment group allocation.

##### Adherence to treatment

ROs will telephone parent/carers at day 3 and weeks 1–3, and 5-11 post treatment allocation, or offer a clinic of home visit, to support correct nasal balloon autoinflation technique, capture adherence data, and promote ongoing treatment adherence. Participants additionally complete a take-home daily autoinflation adherence diary, by placing a sticker in the diary each time the device is used. Reported treatment adherence will be the percentage of maximum treatment adherence for the treatment period.

##### Cost-effectiveness and quality of life

The cost-effectiveness of nasal balloon autoinflation compared to usual care will be measured using the incremental cost-effectiveness ratio (ICER). Although quality-adjusted life years (QALYs) is often used to measure cost-effectiveness, given the nature of the condition and the relatively short follow-up time of the trial, it is unlikely that QALYs will provide a useful measure of quality of life. Therefore, cost-effectiveness will be determined by the ear-related quality of life measured with the OMQ-14 questionnaire at baseline (day 0) and 1 month. The OMQ14 is a 14-question survey. For the purposes of research, scores are calculated using a weighted scoring system, with high scores indicating poorer outcomes. Medical costs, including medication and health service use, and non-medical costs, including from events related to OME and its treatment, will be calculated from data collected during trial visits. Missing cost-effectiveness data will be managed through imputation, rather than deleting observations. Sensitivity analysis will be performed using both one-way and multi-way Monte Carlo simulations.

##### Process evaluation

A process evaluation will be conducted by collecting and analysing data from interviews and written records of research meetings using a qualitative approach. Data will include minutes from the steering committee, investigator team, and community advisory groups meetings and semi-structured interviews with parent/carers and Aboriginal health service-based healthcare providers and research officers. This will allow increased understanding of the outcomes of INFLATE, as well as of the implementation of the trial and of how it is received, and allow increased understanding of trial results [39]. For the process evaluation, a framework approach to analysis will be used to promote a collaborative approach to analysing qualitative data which is useful in research teams with varying experience of conducting qualitative research [40]. In addition to the process evaluation, qualitative exploration of particular aspects of interest within the trial will be undertaken using the qualitative data generated in the process evaluation, with analysis methods determined by the research question for substudies.

The process evaluation of the INFLATE trial is being undertaken together with the WATCH trial as they run parallel in most sites, share common governance, and both examine otitis media management options [33]. This will allow a longitudinal understanding of the implementation and reception of both these trials.

### Statistical analyses

#### Primary outcome

The primary analysis will be on an intention-to-treat basis (ITT), including all children randomised using alpha = 0.05. Secondary analyses will also include per protocol and treatment received (reported by parents) analyses. Sensitivity analysis will be conducted, in which missing data will be handled using multiple imputation in order to avoid underestimation of variance known to be present in single imputation approaches [41]. The relative effect of autoinflation on the primary outcome at 1 month will be estimated using a generalised linear model for binary data with log-link function, reporting relative risks (RR) and 95% confidence intervals. Both unadjusted and adjusted RRs will be reported. Adjustment covariates will include age, sex, whether unilateral or bilateral OME at presentation, and randomisation site. Subgroup analyses of the primary outcome will also be performed by treatment reported by parents, age, and tympanogram type. The unit of analysis will be the participant, rather than the ear, although a secondary analysis will be carried out using each ear as the outcome using generalised estimating equations to account for correlation and clustering.

#### Secondary outcomes

Generalised linear models will also be used to model secondary outcomes, with log-link function used for binary outcomes, identity link for continuous outcomes, and logarithm link for count outcomes. Secondary analyses will include per protocol and treatment received analyses. Missing data will be handled using multiple imputation, using multiple regression models approach. Separate ear-based analyses will be carried out using each ear as the unit of analysis, with generalised estimating equations (GEE) used to account for the correlation and clustering of ears within an individual. Resolution at the ear level will be deemed by a change from type B to type A or C1 at 1 month.

#### Participant retention

Patient enrolment (written informed consent obtained), randomisation, early cessation of treatment, early withdrawal, or loss to follow-up will be reported as a portion of the total number of patients enrolled. Reasons for early cessation of treatment or withdrawal will also be reported.

#### Interim analyses/stopping rules

The trial design may be modified, or the trial suspended or terminated on the grounds of safety such as a life-threatening or fatal serious adverse event (SAE), an unusual cluster of SAEs, or emerging evidence from other studies indicating the trial is unsafe. Suspension or early termination of the trial based on pre-established statistical criteria (efficacy data) to determine whether trial objectives have been met are not being employed in this trial.

### Publication and dissemination

All publications and presentations arising from INFLATE are and will be conducted and reported on in a manner supporting Aboriginal and Torres Strait Islander self-determination, and that is constructive, and respectful of the autonomy of individual Aboriginal Health Services and the communities they serve. In addition to ongoing communication with the community via community reference groups, a final trial report will be generated in lay language and disseminated to the community through the Aboriginal Health Services.

## Discussion

This trial will make an important contribution to the evidence base for the safe and effective management of OME in 3–16-year-old Aboriginal and Torres Strait Islander children. Australian guidelines recommend the use of nasal balloon autoinflation for the treatment of OME in children [29], despite limited evidence of efficacy [30]. There is no evidence to support its use among Aboriginal and Torres Strait Islander children. The INFLATE trial will fill this gap and provide evidence regarding the efficacy, safety, and cost-effectiveness of this treatment alternative in this population. The trial will assess the acceptability of nasal balloon autoinflation compared with usual care with parents and carers, health care providers, and community representatives. INFLATE will report experiences of the trial implementation and governance to inform future randomised controlled trials and research in Aboriginal Health Service settings. The INFLATE trial aims to promote multidirectional learning about ear disease, its impact on hearing, learning, and development, as well as the value of clinical trial co-design and shared Governance in the Aboriginal Health Service setting.

## Trial status

At commencement of recruitment to the trial on 21 December 2017, the INFLATE protocol (version 2.0 dated 26/10/2017) described the inclusion of patients with only bilateral OME and primary outcome assessment at 3 months in accordance with standard care OME treatment guidelines at the time. The protocol has since been amended (version 3.0 dated 26/09/2018) to include children with unilateral or bilateral OME, and primary outcome assessment at 1 month. This amendment was initiated in response to slower than anticipated recruitment, and to minimise loss to follow-up and improve adherence to treatment enabling better determination of treatment efficacy, at least in the short term. The change in protocol conforms with similar studies [30, 31]. As at January 2022, 137 patients of the estimated sample size of 400 have been randomised. The unanticipated COVID-19 pandemic has resulted in a significant reduction in children attending health services face to face, and a diversion of human resources to COVID-19 mitigation activities. This has significantly impacted on the ability of health services to recruit participants during 2020 and 2021. The trial is scheduled to cease recruitment on 31 December 2022.

## Supplementary Information

**Additional file 1.** SPIRIT 2013 Checklist.

## Data Availability

The INFLATE protocol details the extent of and who can access data during the trial. The protocol states an agreement between the WSU and health services is required before services can commence recruitment. It is within this agreement that access and limits to and ownership of the final data set is defined. Consent to share the final dataset with the broader scientific community was not sought from participating sites and participants at the time of initiation of this trial. Data sharing requests will be managed after being directed to the Steering Committee in the first instance and the corresponding author thereafter. Public access to the full protocol and supporting documentation, including the participant information sheet and consent forms, participant-level dataset, and statistical code, will be granted on reasonable request.

## Abbreviations

AI: Associate investigator
AOM: Acute otitis media
CI: Chief investigator
DSMB: Data Safety Monitoring Board
GP: General Practitioner
HREC: Human Research Ethics Committee
IVRS: Interactive Voice Response System
OM: Otitis media
OME: Otitis media with effusion
OMQ-14: Ear-health related Quality of Life questionnaire
QALYs: Quality-adjusted life years
WSU: Western Sydney University
